# High-Throughput Identification of Epigenetic Compounds to Enhance Chicken Host Defense Peptide Gene Expression

**DOI:** 10.3390/antibiotics11070933

**Published:** 2022-07-12

**Authors:** Zhuo Deng, Wentao Lyu, Guolong Zhang

**Affiliations:** 1 Department of Animal and Food Sciences, Oklahoma State University, Stillwater, OK 74078, USA; zhuo.deng@okstate.edu (Z.D.); lvwt@zaas.ac.cn (W.L.); 2 State Key Laboratory for Managing Biotic and Chemical Threats to the Quality and Safety of Agro-Products, Institute of Agro-Product Safety and Nutrition, Zhejiang Academy of Agricultural Sciences, Hangzhou 310021, China

**Keywords:** antimicrobial resistance, antibiotic alternatives, host defense peptides, host defense peptide inducers, high throughput screening, histone deacetylase inhibitors, entinostat, chickens

## Abstract

Enhancing the synthesis of endogenous host defense peptides (HDPs) has emerged as a novel antibiotic-free approach to infectious disease control and prevention. A number of epigenetic compounds have been identified as HDP inducers and several have proved beneficial in antimicrobial therapy. However, species-specific regulation of HDP synthesis is evident. In attempt to identify epigenetic compounds with potent HDP-inducing activity for poultry-specific application, we developed a stable luciferase reporter cell line, known as HTC/*AvBD10-luc*, following our earlier construction of HTC/*AvBD9-luc*. HTC/*AvBD10*-*luc* was developed through permanent integration of a chicken macrophage cell line, HTC, with a lentiviral luciferase reporter vector driven by a 4-Kb *AvBD10* gene promoter. Using a high throughput screening assay based on the two stable cell lines, we identified 33 hits, mostly being histone deacetylase (HDAC) inhibitors, from a library of 148 epigenetic compounds. Among them, entinostat and its structural analog, tucidinostat, were particularly effective in promoting multiple HDP gene expression in chicken macrophages and jejunal explants. Desirably, neither compounds triggered an inflammatory response. Moreover, oral gavage of entinostat significantly enhanced HDP gene expression in the chicken intestinal tract. Collectively, the high throughput assay proves to be effective in identifying HDP inducers, and both entinostat and tucidinostat could be potentially useful as alternatives to antibiotics to enhance intestinal immunity and disease resistance.

## 1. Introduction

Use of antibiotics in the feed has been found to promote the growth of livestock animals since the 1940s [1,2]. However, widespread use of antibiotics has led to the emergence of antibiotic-resistant pathogens, which has become a serious threat to public health worldwide [3,4]. Therefore, nontherapeutic use of antibiotics has been phased out for the livestock production in the European Union, U.S., and a growing list of countries [5]. Finding effective alternatives of antibiotics is thus critically important to ensure animal health and production efficiency [6,7]. 

Host defense peptides (HDPs) are a critical component of the first-line defense against infections [8,9]. In vertebrate animals, HDPs mainly belong to either the cathelicidin or defensin family, which are synthesized by the circulating leukocyte, skin keratinocytes as well as mucosal epithelial layer of digestive, urogenital, and respiratory tracts [8,9,10]. Humans produce one cathelicidin (*LL-37*), six α-defensins, one θ-defensin, and scores of β-defensins [11], while the chicken genome encodes a total of 4 cathelicidins (*CATH1*-*3* and *CATHB1*) and 14 avian β-defensins (*AvBD1-14*) [12,13]. In addition to inflammation and infection, HDPs have been found to be transcriptionally regulated by a large group of small-molecule compounds such as vitamin D_3_ and lactose [14,15,16]. Epigenetic compounds, particularly histone deacetylase (HDAC) inhibitors such as butyrate, are effective HDP inducers [17,18]. In fact, several HDP inducers have been experimentally verified to be effective in protecting animals from infections [14,17,18].

Although many HDP inducers appear to be capable of regulating HDP gene expression across multiple animal species, a few are effective only in certain species. For example, vitamin D_3_ is highly efficient in inducing human cathelicidin expression, but loses its activity in mice, because of a lack of vitamin D response element in the mouse cathelicidin gene promoter [19]. Vitamin D_3_ also appeared to be very weak in HDP induction in chickens [20]. It is also apparent that HDP genes are differentially regulated and that the same HDP genes may be regulated differently in different cell types of an animal species [21,22]. Therefore, it is beneficial to identify species-specific compounds that work efficiently in simultaneous induction of multiple HDP genes across multiple cell types. 

To identify potent HDP inducers specifically for poultry applications, we developed a robust cell-based high throughput screening (HTS) assay by placing the luciferase reporter gene under the control of a chicken HDP gene promoter. We recently described the development of a stable luciferase reporter cell line known as HTC/*AvBD9-luc*, with permanent integration of a luciferase gene driven by a 2-Kb *AvBD9* gene promoter [23]. Although it is readily inducible by many HDP inducers, *AvBD9* is lowly expressed, while *AvBD10* is among the HDP genes that are most abundantly expressed in the chicken intestinal tract [24,25]. To identify small-molecule compounds that are capable of directly modulating *AvBD10* gene expression, we constructed a stable luciferase reporter cell line known as HTC/*AvBD10-luc* under the control of a 4-Kb promoter sequence of the *AvBD10* gene. Both HTC/*AvBD9-luc* and HTC/*AvBD10-luc* cells were used in screening a library of small-molecule epigenetic compounds. Through a series of primary and secondary screening, we identified two HDAC inhibitors, entinostat and tucidinostat, that are highly potent in inducing the expression of multiple chicken HDP genes. Coupled with their anti-inflammatory activity, both compounds have the potential to be further explored as alternatives to antibiotics for applications in poultry and possibly other animal species. 

## 2. Results

### 2.1. Establishment of an HTS Assay to Identify AvBD9 and AvBD10 Inducers

**Figure 1:**
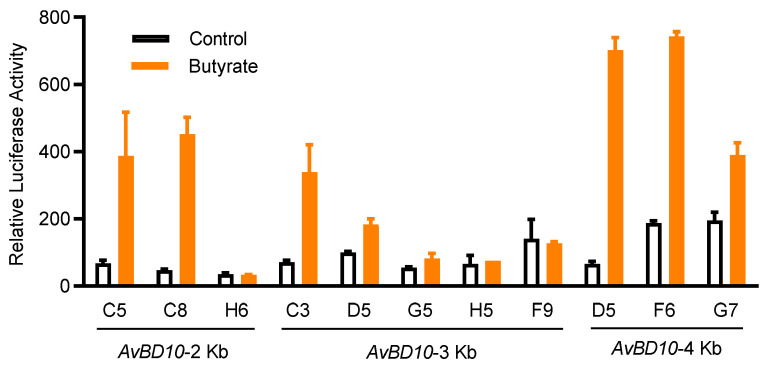
Relative luciferase activities of HTC/*AvBD10-luc* stable cell clones in response to butyrate. Individual cell clones were integrated with a lentiviral luciferase reporter vector driven by *AvBD10* gene promoter segments of approximately 2-, 3-, and 4-Kb. Each cell clone was stimulated in duplicate with or without 16 mM butyrate for 24 h. The luciferase activity was measured relative to the background luciferase activity of an equal volume of the cell culture medium, as an indication of *AvBD10* gene activation. The results are means ± SEM of two independent experiments.

We previously established a stable chicken luciferase reporter cell line known as HTC/*AvBD9-luc* [23]. Using a similar approach, we developed several stable cell clones with permanent integration of a luciferase reporter gene driven by three different *AvBD10* promoter constructs. Among multiple HTC/*AvBD10-luc* cell clones obtained after limiting dilution, clone D5 gave the highest fold induction in response to 16 mM butyrate, with a minimum background (Figure 1), and therefore, was selected for subsequent HTS assays. When 16 mM butyrate was used as the positive control, Z’-factor of the HTS assay was determined to be 0.62, which is considered to be excellent [26]. 

**Figure 2:**
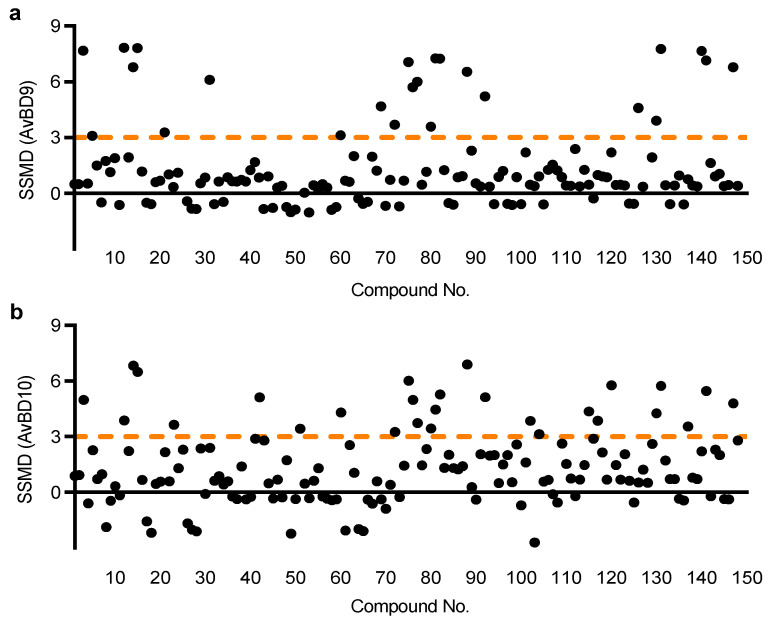
High throughput screening for HDP inducers. Two stable luciferase reporter cell lines, HTC/*AvBD9-luc* (**a**) and HTC/*AvBD10-luc* (**b**), were employed to screen a library of 148 epigenetic compounds at the final concentration of 20 µM each. Relative luciferase activity was measured following a 24-h exposure and normalized to the cytotoxicity of each compound, followed by calculation of the strictly standardized mean difference (SSMD) value. Those with a minimum SSMD value of 3 were identified as the hits.

**Figure 3:**
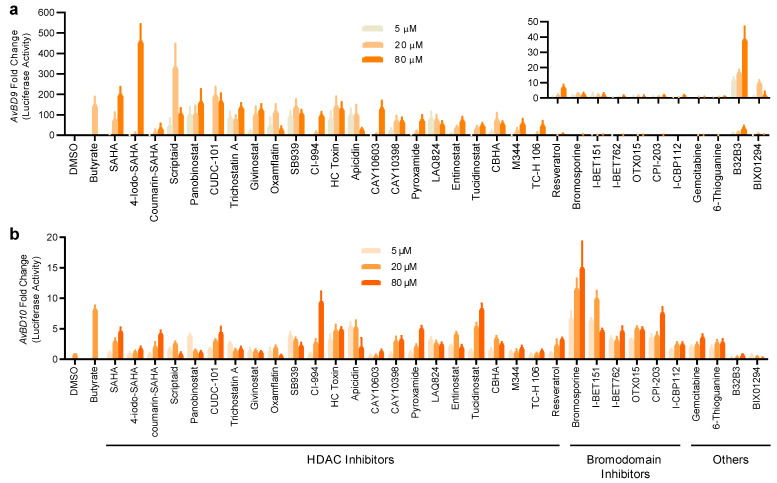
Concentration-dependent activation of the *AvBD9* and *AvBD10* gene promoters by 33 epigenetic compounds. HTC/*AvBD9-luc* (**a**) and HTC/*AvBD10-luc* (**b**) luciferase reporter cell lines were stimulated in duplicate with three different concentrations (5, 20, and 80 μM) of each for 24 h, followed by luciferase and cytotoxicity assays. Butyrate (16 mM) and an equal volume of DMSO were used as positive and negative controls, respectively. Fold changes were calculated as relative luciferase activity of a compound divided by that of DMSO-treated cells, after normalization to cell viability. The inset in Panel (**a**) shows the fold changes in the *AvBD9* promoter-driven luciferase activity of non-HDAC inhibitors. The results are means ± SEM of three independent experiments.

Both HTC/*AvBD9-luc* and HTC/*AvBD10-luc* cell lines were employed sequentially to screen an Epigenetics Screening Library (Cayman Chemical, Ann Arbor, MI, USA) containing 148 compounds. Using a strictly standardized mean difference (SSMD) value of 3.0 as the threshold, which is considered to be strongly positive [27], for either HTC/*AvBD9-luc* or HTC/*AvBD10-luc* cells, a non-redundant list of 33 compounds were identified (Figure 2). A number of compounds also had an SSMD value between 1 and 3 (Figure 2), and can be considered moderately or fairly strong hits [27]. Interestingly, none caused a significant inhibition as evidenced by a lack of an SSMD value of <−3 (Figure 2). Among 33 very strong hits, 23 are HDAC inhibitors and 6 are bromodomain inhibitors, while the remaining 4 belong to other classes of epigenetic compounds (Figure 3).

To further verify their HDP-inducing ability, 33 hits were applied to both stable cell lines at 3 different concentrations (5, 20, and 80 μM) for 24 h. It is obvious that HDAC inhibitors are highly potent in increasing the luciferase activity in a concentration-dependent manner in both reporter cell lines (Figure 3), except for resveratrol, which has HDAC inhibitory activities and other epigenetic functions [28]. Six bromodomain inhibitors were weak *AvBD9* inducers, but among the most potent *AvBD10* inducers (Figure 3). Similarly, two nucleoside analogs, gemcitabine and 6-thioguanine, strongly induced *AvBD10*, but not *AvBD9*. B32B3 and BX01294 were also identified as weak HDP inducers (Figure 3).

### 2.2. Validation of HDP Inducers in Chicken HTC and HD11 Macrophage Cell Lines

**Figure 4:**
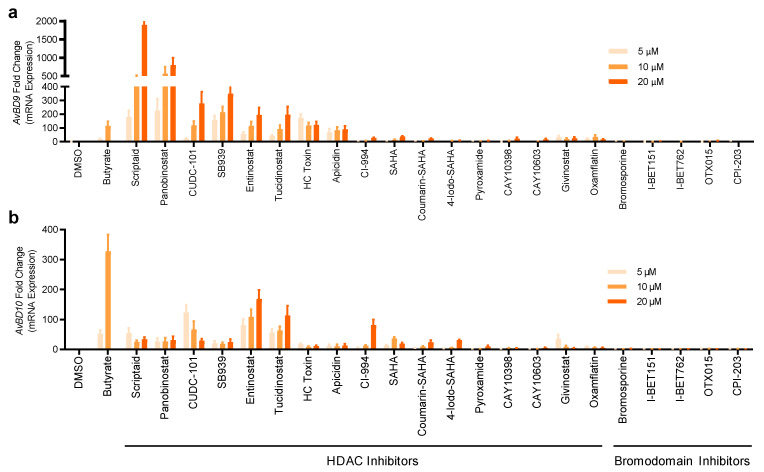
Induction of the *AvBD9* and *AvBD10* mRNA expression by 22 selected compounds in chicken HTC macrophages. HTC cells were stimulated in duplicate with three concentrations (5, 10, and 20 μM) of each compound for 24 h, followed by RT-qPCR analysis of the *AvBD9* (**a**) and *AvBD10* (**b**) mRNA expression. HTC cells treated with 2- and 4-mM butyrate were used as positive controls. Fold changes in HDP mRNA expression were calculated relative to DMSO-treated cells using the ΔΔCt method. The results are means ± SEM of 3–4 independent experiments.

To directly validate the ability of the hits to induce chicken HDP mRNA expression, parental HTC cells were further stimulated with 22 selected compounds that were strong in activating *AvBD9* and *AvBD10* genes. Induction of the mRNA expression levels for *AvBD9* and *AvBD10* was evaluated by reverse transcription-quantitative PCR (RT-qPCR). All compounds were capable of enhancing either or both *AvBD9* and *AvBD10* mRNA expression in a concentration-dependent manner (Figure 4). Seven HDAC inhibitors including scriptaid, panobinostat, CUDC-101, SB939, entinostat, tucidinostat, and HC toxin were effective in inducing the expressions of both *AvBD9* and *AvBD10* genes in HTC cells, and entinostat and tucidinostat appeared to be among the most potent HDAC inhibitors in *AvBD10* mRNA induction (Figure 4). Apparently, a gene-specific induction pattern was obvious in response to HDAC inhibitors. For example, scriptaid and panobinostat were highly efficient in triggering the gene expression of *AvBD9*, but not *AvBD10* (Figure 4).

**Figure 5:**
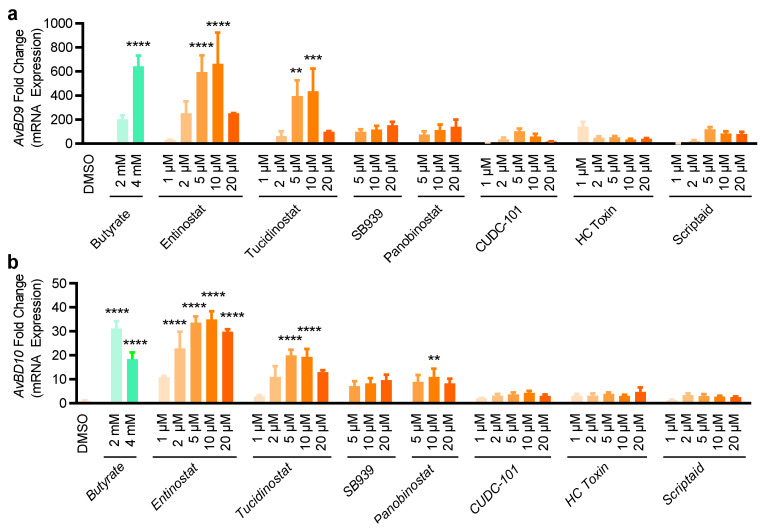
Induction of the *AvBD9* and *AvBD10* mRNA expression by seven selected compounds in chicken HD11 macrophages. HD11 cells were stimulated in duplicate with indicated concentrations of each compound for 24 h, followed by RT-qPCR of *AvBD9* (**a**) and *AvBD10* (**b**) expression. Fold changes were calculated relative to DMSO-treated cells using the ΔΔCt method. The results are means ± SEM of 3–4 independent experiments. ** *p* < 0.01, *** *p* < 0.001, and **** *p* < 0.0001 relative to the DMSO control (by one-way ANOVA and post-hoc Dunnett’s multiple comparisons test).

All five selected bromodomain inhibitors showed a marginal effect on the induction of both *AvBD9* and *AvBD10* mRNA (Figure 4). Therefore, seven HDAC inhibitors were further chosen to examine for their HDP-inducing activity in a different chicken macrophage cell line, HD11 [29]. Although all seven compounds increased both *AvBD9* and *AvBD10* mRNA expression in HD11 cells after 24 h, entinostat and tucidinostat were obviously more efficient than the others (Figure 5). Cell-specific induction of HDP mRNA expression was also evident. While scriptaid, panobinostat, and SB939 were among the most potent compounds in inducing *AvBD9* gene expression in HTC cells (Figure 4), but lost much of their *AvBD9*-inducing capacity in HD11 cells (Figure 5).

**Figure 6:**
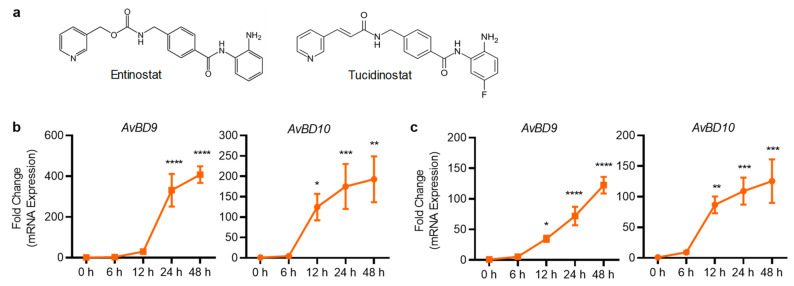
Time-dependent induction of *AvBD9* and *AvBD10* mRNA expression in chicken HTC cells in response to structurally-related entinostat and tucidinostat (**a**). HTC cells were stimulated in duplicate with 10 µM entinostat (**b**) or tucidinostat (**c**) for 6, 12, 24, or 48 h, followed by RT-qPCR analysis. The results are means ± SEM of 2–3 independent experiments. * *p* < 0.05, ** *p* < 0.01, *** *p* < 0.001, and **** *p* < 0.0001 relative to the non-stimulation control (by one-way ANOVA and post-hoc Dennett’s multiple comparisons test).

Both entinostat and tucidinostat are structurally related, with the latter carrying a fluorine group that is reported to be more stable and have a longer half-time than entinostat [30] (Figure 6a). Both compounds were selected for further analysis because they are highly effective in inducing *AvBD9* and *AvBD10* gene expression in both chicken HTC and HD11 cells. Stimulation of HTC cells with 10 μM entinostat (Figure 6b) or tucidinostat (Figure 6c) for 6, 12, 24, and 48 h revealed a time-dependent increase in both *AvBD9* and *AvBD10* gene expression, with 24 and 48 h approaching the peak induction. 

### 2.3. Entinostat and Tucidinostat Enhance Multiple HDP Gene Expression in Jejunal Explants

**Figure 7:**
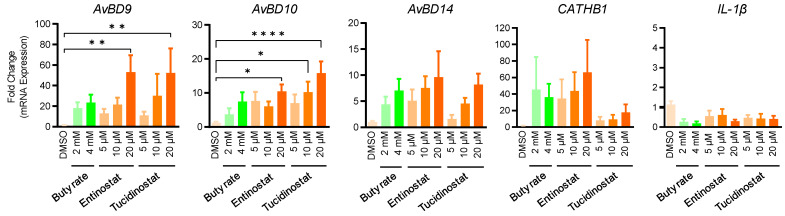
Regulation of HDP and *IL-1β* gene expression in chicken jejunal explants by entinostat and tucidinostat. Jejunal explants were stimulated in triplicate with or without indicated concentrations of either compound for 24 h, followed by RT-qPCR analysis. DMSO was added as a negative control. The results are means ± SEM of 3 independent experiments. * *p* < 0.05, ** *p* < 0.01, **** *p* < 0.0001 (by one-way ANOVA and post-hoc Dunnett’s multiple comparisons test).

In order to evaluate the efficacy of entinostat and tucidinostat in enhancing HDP expression in the intestinal tract, chicken jejunal explants were prepared and treated with different concentrations of both compounds for 24 h. Besides *AvBD9* and *AvBD10*, we also analyzed the expression of *AvBD14*, *CATHB1*, and interleukin-1β (*IL-1β*) by RT-qPCR. A concentration-dependent increase in *AvBD9*, *AvBD10*, and *AvBD14* was observed in response to entinostat or tucidinostat (Figure 7). Both compounds at 20 µM significantly induced *AvBD9* and *AvBD10* mRNA expression (*p* < 0.05) and appeared to be more potent than 2- or 4-mM butyrate. Entinostat also showed a concentration-dependent increase in *CATHB1* expression. Desirably, similar to butyrate, neither entinostat nor tucidinostat triggered *IL-1β* gene expression (Figure 7).

### 2.4. Entinostat Increases HDP Expression in Chickens

**Figure 8:**
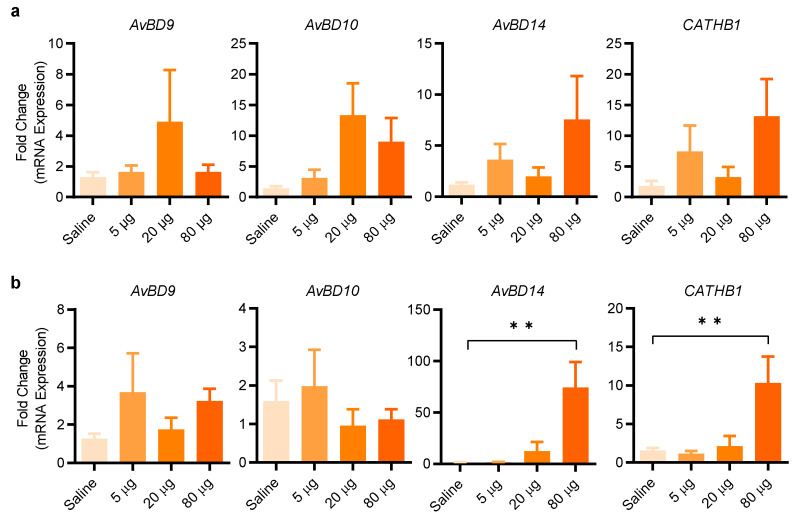
Entinostat-mediated induction of HDP gene expression in the crop and jejunum of chickens. Three-day-old broiler chickens were inoculated with or without an indicated amount of entinostat in 0.5 mL saline by oral gavage every 12 h for 2 days, with 12 animals per treatment. A segment of the crop (**a**) and jejunum (**b**) was collected from each animal for RT-qPCR analysis of HDP mRNA expression (n = 12). ** *p* < 0.01 (by one-way ANOVA and post-hoc Dunnett’s multiple comparisons test).

To further evaluate the efficacy of entinostat in enhancing HDP gene expression in live animals, we administered three different doses (5, 20, and 80 µg/animal) of entinostat by oral gavage twice a day for two days. Analysis of the crop of the chickens revealed 20 µg/animal to be optimal in *AvBD9* and *AvBD10* expression, while a further increase to 80 µg/animal resulted in a diminished induction of both HDP genes (Figure 8a). On the other hand, a maximum induction of *AvBD14* and *CATHB1* in the crop by entinostat was observed at 160 µM (Figure 8a). In the jejunum, *AvBD9* and *AvBD10* were minimally induced by entinostat; however, 80 µg/animal gave a significant induction in both *AvBD14* and *CATHB1* expression (*p* < 0.01) (Figure 8b). The results indicated that gene- and tissue-specific induction of HDP genes by entinostat is evident. 

## 3. Discussion

Epigenetics plays an important role in gene regulation [31]. Acetylation and deacetylation of histones are among the major epigenetic mechanisms that regulate gene expression and consequently, various physiological processes [32]. Acetylation of the lysine residues on histone tails often leads to chromatin relaxation and enhanced transcription, while deacetylation incur an opposite effect [32]. Histone acetylation and deacetylation are achieved through separate groups of enzymes known as histone acetyltransferases (HATs) and histone deacetylases (HDACs), respectively [32]. While the CREB-binding protein (CBP)/EP300 family are well-known HATs, four classes of HDACs consisting of over a dozen proteins exist [32,33]. A large group of structurally diverse compounds have been identified to inhibit either nearly all HDACs or specific classes of HDACs [33]. Bromodomain is a structural motif that recognizes acetylated lysine residues of proteins including histones, and a dozen bromodomain-containing proteins have been discovered and found to participate actively in chromatin remodeling and transcriptional regulation [34]. Bromodomain inhibitors have shown therapeutic benefits in treating inflammatory disorders and different types of cancer [34,35]. A variety of HDAC inhibitors were recently found to induce HDP gene expression [17], while the involvement of bromodomain inhibitors in regulating HDP expression is yet to be reported.

In this study, we developed a cell-based, chicken HDP gene promoter-driven HTS luciferase assay to screen for small-molecule epigenetic compounds with the ability to induce endogenous HDP synthesis. We chose the chicken *AvBD9* gene promoter because *AvBD9* is the most readily regulated HDP genes in response to butyrate and many other compounds [21]. *AvBD10* was selected because it is among the most abundantly expressed HDPs in the intestinal tract of chickens [24,25]. Therefore, identifying the compounds that are potent in regulating both *AvBD9* and *AvBD10* genes could be potentially explored to promote chicken intestinal immunity and replace in-feed antibiotics. Among 148 epigenetic chemicals, HDAC inhibitors are clearly the most potent in HDP gene induction, which is consistent with recent high-throughput screening efforts in humans [36] and pigs [37]. However, HDP induction efficiency of HDAC inhibitors is not strictly in linear relationship with their activity of inhibiting histone deacetylase [38,39]. For example, trichostatin A (TSA) is much more potent than butyrate in HDAC inhibition; however, it is not among the most efficacious chemicals identified in our study.

Here, we have identified entinostat and its structural analog, tucidinostat, as the most potent compounds across several cell types through a series of the in vitro, ex vivo, and in vivo evaluations. In many cases, entinostat and tucidinostat are more potent than butyrate. Entinostat, also known as MS-275 and SNDX 275, and tucidinostat, also known as chidamide, are class I HDAC inhibitors [33]. Consistently, entinostat was recently identified as a potent HDP inducer in humans [40] and shown to protect rabbits from an experimental cholera [41]. In this study, we observed a significant increase in the multiple HDP gene expression in the intestinal tract of the chickens following oral administration of entinostat, suggesting that entinostat is an effective HDP inducer that works not only in humans, but also in chickens, unlike some of the other HDP inducers such as vitamin D_3_ that works in only limited species [19]. Desirably, entinostat induces HDP synthesis without triggering inflammation, as seen in chicken jejunal explants, which is consistent with many of these HDAC inhibitors [42]. However, it is noted that entinostat or tucidinostat was not identified as top hits in inducing porcine HDP gene expression following high throughput screening of the same epigenetic compound library [37]. Additional studies are warranted to evaluate the HDP-inducing potency and, more importantly, the efficacy in disease control and prevention of entinostat or its structural analogs in different animal species.

In addition to HDAC inhibitors, we have discovered several bromodomain inhibitors to be capable of inducing HDP gene expression, albeit with a modest activity. To our knowledge, this is the first report on the HDP-inducing activity for this group of compounds in any species. Among six bromodomain inhibitors identified in this study, bromosporine, I-BET151, I-BET762, OTX015, and CPI-203 are pan-inhibitors of bromodomain-containing proteins [34,35], while I-CBP112 is more specific to CBP/EP300 [43]. Additionally, two nucleoside analogs, namely gemcitabine and 6-thioguanine, have a weak HDP-inducing activity as well. Both compounds work mainly by incorporating into DNA and RNA as fraudulent bases to cause termination of cellular synthesis of DNA/RNA and have been approved for treatment of cancers and inflammatory bowel diseases [44,45]. 

BIX01294, a histone methyltransferase inhibitor [46], has also shown to be active in HDP induction, which is consistent with our earlier observation [47]. In the same study, BIX01294 was further demonstrated to synergize strongly with HDAC inhibitors to induce chicken HDP gene expression [47]. B32B3, a compound that blocks VprBP-mediated phosphorylation of histone 2A [48], is also a weak HDP inducer. Identification of different classes of epigenetic compounds with the HDP-inducing activity has provided new options for host-directed antimicrobial therapy, although the mechanism by which many of these compounds trigger HDP gene expression remains largely unexplored. Because of our earlier demonstration that, albeit being weak alone, histone methyltransferase inhibitors and DNA methyltransferase inhibitors are synergistic with HDAC inhibitors in HDP gene induction [47], suggesting the potential to explore the combinations of different classes of epigenetic compounds in antimicrobial therapy. 

Although the newly-developed HTS assay has proved to be effective in discovering HDP inducers, we observed a discrepancy between the HTS assay of the luciferase activity and RT-qPCR assay of HDP mRNA expression. Prominent examples are those bromodomain inhibitors that show a strong activity in the HTS luciferase assay (Figure 3), but lose much of the activity in inducing *AvBD10* mRNA expression (Figure 4). The reason is likely due to the fact that the luciferase assay is based on the ability of a compound to activate a 4-Kb *AvBD10* gene promoter fragment, while RT-qPCR measures the mRNA expression levels of the native *AvBD10* gene. It is possible that certain negative *cis*-regulatory elements exist upstream of the 4-Kb *AvBD10* gene promoter. Without them, *AvBD10* gene activation becomes more pronounced in response to bromodomain inhibitors as evidenced by heightened luciferase activities. In addition to those bromodomain inhibitors, several HDAC inhibitors showed a discrepancy in relative potency between the luciferase activity and their ability to induce HDP mRNA expression. The reason is unclear, but it is likely that some compounds may regulate certain transcription factors that bind beyond the targeted promoter regions of *AvBD9* and *AvBD10* genes. 

Taken together, we have discovered a number of epigenetic compounds capable of inducing chicken HDP gene expression. Two HDAC inhibitors, entinostat and tucidinostat, are particularly potent to induce endogenous HDP expression without triggering an inflammatory response. Therefore, both compounds have potential as attractive candidates as alternatives to antibiotics for applications in poultry and possibly other animals including humans. Additionally, the HTS assay developed in this study can be utilized to identify additional HDP inducers. This approach may also be adapted for the discovery of HDP inducers in other species including humans. 

## 4. Materials and Methods

### 4.1. Ethics Statement

All animal experiments in this study were approved by the Institutional Animal Care and Use Committee of Oklahoma State University under protocol no. AG1610. 

### 4.2. Cell Culture Media and Chemical Reagents 

Cell culture medium RPMI 1640, penicillin, streptomycin, and gentamicin were all purchased from Hyclone (Logan, UT, USA), and fetal bovine serum (FBS) was procured from Atlanta Biologicals (Lawrenceville, GA, USA). Sodium butyrate was purchased from MilliporeSigma (St. Louis, MO, USA), while all other individual compounds were acquired from Cayman Chemical (Ann Arbor, MI, USA). An epigenetics screening library containing 148 small-molecule epigenetic compounds was obtained from Cayman Chemical. A bulk amount of entinostat was also procured from MedChemExpress (Monmouth Junction, NJ, USA) for animal trials.

### 4.3. Construction of Luciferase Reporter Vectors Driven by Chicken HDP Gene Promoters

*AvBD10* gene promoter was cloned from chicken genomic DNA using Advantage 2 PCR Kit (Takara Bio USA, San Jose, CA, USA). Three *AvBD10* gene promoter segments of approximately 2-, 3-, and 4-kb were amplified with a common reverse primer (tac acg cct aac tag tAG TTG TGG ACT GCG TGC CCC A) and three unique forward primers (ttt tat cga tga att cGC TCT GCT CTC AGG GCA TTC T, ttt tat cga tga att cAG TCC ATG TTC TTT CAT CTG G, ttt tat cga tga att cCA ACC ATC ATG TGT ATG TAG G), respectively. The primer sequences in upper case are gene-specific, while the sequences in lower case were included for subsequent cloning of the PCR products into a lentiviral vector pGreenFire1 (pGF1) (System Biosciences, Palo Alto, CA, USA) predigested with EcoRI and SpeI (Promega, Madison, WI, USA) using In-Fusion HD Cloning Kit (Takara Bio USA). After bacterial transformation, recombinant vectors were confirmed for the presence of appropriate AvBD10 gene promoter sequences by Sanger sequencing. 

### 4.4. Development of Stable Luciferase Reporter Cell Lines

Stable cell lines were developed by infecting a chicken macrophage cell line, HTC [49], with pseudo-lentiviruses containing *AvBD10*-driven luciferase reporter gene. To produce pseudoviruses, 1 × 10^6^ HEK 293T cells were seeded into a 60-mm cell culture plate in complete DMEM containing 10% FBS, 100 U/mL penicillin, and 100 µg/mL streptomycin overnight prior to transfection with 3 µg of each recombinant reporter vector and a mixture of 3 µg of lentiviral packaging plasmids consisting of pMD2.G, pRSV-REV, and pMDLg/pRRE (System Biosciences, Palo Alto, CA, USA) using Lipofectamine 3000 Reagent (Thermo Fisher Scientific, Waltham, MA, USA). After overnight incubation, transfected cells were replenished with fresh complete DMEM. The pseudoviruses were harvested from the cell culture medium after another 48 h. To infect with pseudoviruses, 1 × 10^5^ HTC cells were seeded into a 6-well tissue culture plate containing 2 mL RPMI 1640 medium supplemented with 10% FBS overnight prior to inoculation with 200 μL pseudoviruses with addition of polybrene (Santa Cruz Biotechnology, Dallas, TX, USA) at the final concentration of 5 μg/mL. 

After 24 h incubation, cells were replenished with fresh complete RPMI 1640 medium containing 10% FBS, 100 U/mL penicillin, 100 µg/mL streptomycin, and 0.5 μg/mL of puromycin (Thermo Fisher Scientific) for selection of stably integrated cell clones for two weeks. The cells were replenished with fresh complete RPMI 1640 medium containing 0.5 μg/mL puromycin every 2–3 days during antibiotic selection. Individual stable cell clones were obtained by limiting dilution of cells at 0.1 and 1 cell/well in 96-well tissue culture plates in the presence of 0.5 μg/mL puromycin. Single cell clones were retrieved from individual wells after becoming 20–30% confluent, gradually expanded into 6-well and 10-cm plates for stocking and further characterization.

### 4.5. Optimization and Characterization of the HTS Assay

To identify the *AvBD10* gene promoter construct that is the most responsive to butyrate, individual cell clones were seeded at 2 × 10^4^ cells/well overnight in 50 μL of complete RPMI 1640 medium in a 96-well tissue culture plates, followed by stimulation with or without 16 mM sodium butyrate for another 24 h. The luciferase activity was measured on a Modulus Single Tube Luminometer (Turner Biosystems, Sunnuvale, CA, USA) using Steady-Glo^®^ Luciferase Assay System (Promega Corporation, Madison, WI, USA) according to the manufacturer’s instructions. To assess the quality of the HTS assay based on the most responsive HTC/*AvBD10-luc* cell clone D5, cells were seeded in 96-well plates and stimulated with or without 16 mM butyrate in 12 replicate wells for 24 h. The luciferase activity was measured on L-Max II Luminescence Microplate Reader (Molecular Devices, Sunnyvale, CA, USA). The Z’-factor was calculated as $1-\frac{\left({3\sigma }_{p}{+3\sigma }_{n}\right)}{\left|\mu_{p}-{\;\mu }_{n}\right|}$, where σ_p_ and σ_n_ are standard deviations of positive and negative controls, while μ_p_ and μ_n_ are the mean luciferase activity of positive and negative controls, respectively [26]. An HTS assay is considered excellent if 1 > Z’ ≥ 0.5 [26].

### 4.6. HTS Assay for HDP-Inducing Compounds

HTC/*AvBD10-luc* cell clone D5 were seeded at 2 × 10^4^ cells/well in 50 μL of complete RPMI 1640 medium in 96-well plates overnight prior to exposure to 20 µM of each of the 148 compounds in an Epigenetics Screening Library (Cayman Chemical) in individual wells for 24 h prior to luciferase assay. To assess potential cytotoxicity of each compound, an alamarBlue dye (Thermo Fisher Scientific) was added to a final concentration of 10% and incubated for 4 h as we previously described [23,37,50]. Cytotoxicity was assessed in live cells by measuring the fluorescence at 545 nm excitation and 590 nm emission on Fx80 Microplate Fluorescence Reader (BioTek Instruments, Winooski, VT, USA), followed by luciferase assay on L-Max II Luminescence Microplate Reader (Molecular Devices) using Steady-Glo^®^ Luciferase Assay System (Promega). The relative luciferase activity was normalized to the cell viability for each compound as we described [23,37,50]. To select hits, strictly standardized mean difference (SSMD) values were subsequently calculated as $\frac{\left(\log_{2}{µ}_{s}\;-\;\log_{2}{µ}_{n}\right)}{\surd 2\sigma }$, where μ_s_ and μ_n_ are the mean luciferase activity of positive and negative controls, respectively, while σ is the standard deviation of the negative control [27]. A compound is considered as a strongly positive hit with an SSMD value of ≥3 [27]; therefore, all those compounds showing an SSMD value of ≥3 in the primary screening of either HTC/*AvBD10-luc* or HTC/*AvBD9-luc* cells were selected for further dose-response analysis in both stable cell lines at three different concentrations (5, 20, and 80 μM) for 24 h. The luciferase activity of each compound was normalized to its viability for each concentration. All compounds are generally non-cytotoxic with an average cell viability of approximately 95% at 20 μM.

### 4.7. HDP mRNA Induction of the Hit Compounds in Two Chicken Macrophage Cell Lines

Two chicken macrophage cell lines, HTC and HD11, were maintained in complete RPMI 1640 medium containing 10% FBS, 100 U/mL penicillin and 100 µg/mL streptomycin and used to assess the ability of the hits to induce both *AvBD9* and *AvBD10* mRNA expression. Cells were treated in duplicate with different concentrations of each compound in 12-well plates for 24 h before they were lysed in RNAzol^®^ RT Reagent (Molecular Research Center, Cincinnati, OH, USA). Total RNA was then subjected to RT-qPCR analysis of the HDP gene expression as described below. Sodium butyrate was used as the positive control, while an equal volume of dimethyl sulfoxide (DMSO) was used as the negative control. 

### 4.8. Induction of HDP mRNA Expression in Chicken Jejunal Explants

To further determine the ex vivo effect of selected compounds on HDP gene expression, chicken jejunal explants were collected from 1- to 2-week-old Cobb broiler chickens and washed thoroughly in cold phosphate-buffered saline (PBS) containing 100 U/mL penicillin, 100 µg/mL streptomycin, and 100 μg/mL gentamicin. These segments were then cut into small fragments (approximately 0.5 × 0.5 cm^2^) and dispensed individually into 6-well plates containing 4 mL of RPMI 1640 medium supplemented with 20 mM HEPES, 10% FBS, 100 U/mL penicillin, 100 µg/mL streptomycin, and 100 μg/mL gentamicin. After being treated with selected compounds in triplicate, the cells were incubated for 24 h at 37 °C in a hypoxia chamber (StemCell Technologies, Vancouver, QC, Canada) flushed with 95% O_2_ and 5% CO_2_. The explants were then homogenized in RNAzol RT for total RNA isolation and RT-qPCR analysis of chicken HDP gene expression. 

### 4.9. In Vivo Induction of HDP mRNA Expression by Entinostat

To determine the ability of entinostat to induce HDP expression in vivo, newly-hatched male Cobb broiler chicks were obtained from Cobb-Vantress Hatchery (Siloam Springs, AR, USA). After two days of free access to standard diet and water, the chicks were administered with or without 5, 20, or 80 μg entinostat in 0.5 mL saline by oral gavage every 12 h for a total of three times. Twelve hours after the last administration, all animals were euthanized with CO_2_, and a 1-cm segment of the crop and proximal jejunum was collected in liquid nitrogen and homogenized in RNAzol^®^ RT for RNA isolation and HDP gene expression analysis. 

### 4.10. Total RNA Isolation and RT-qPCR

Total RNA was isolated using RNAzol RT by following the manufacturer’s protocol. The first-strand cDNA was synthesized with 0.3 µg of total RNA in 4-µL reactions using iScript cDNA Synthesis Kit (Bio-Rad Laboratories, Hercules, CA, USA). A tenth of the cDNA was subsequently used in 10 µL reactions for qPCR analysis of HDP gene expression on iQ5 Real time PCR Detection System (Bio-Rad Laboratories) using Maxima SYBR Green qPCR Master Mix Kit (Thermo Fisher Scientific) and gene-specific primers, as previously described [51,52,53,54]. Chicken glyceraldehyde 3-phosphate dehydrogenase (*GAPDH*) was used as a reference gene, and HDP genes (*AvBD9*, *AvBD10*, *AvBD14*, and *CATHB1*) and a proinflammatory cytokine gene (*IL-1β*) were amplified. The fold change in gene expression in response to a compound relative to the DMSO was calculated using the ΔΔCt method [55].

### 4.11. Statistical Analysis

Data were analyzed with GraphPad PRISM (GraphPad Software, La Jolla, CA, USA), and represented as means ± SEM. One-way ANOVA was applied, followed by post-hoc Dunnett’s multiple comparisons test. *p* < 0.05 was considered statistically significant.

## Data Availability

All data generated during this study are included in this published article.

## References

[1] Moore P.R., Evenson A., Luckey T.D., McCoy E., Elvehjem C.A., Hart E.B. (1946). Use of sulfasuxidine, streptothricin, and streptomycin in nutritional studies with the chick. J. Biol. Chem..

[2] Jukes T.H., Stokstad E.L.R., Taylor R.R., Cunha T.J., Edwards H.M., Meadows G.B. (1950). Growth-promoting effect of aureomycin on pigs. Arch. Biochem..

[3] Skandalis N., Maeusli M., Papafotis D., Miller S., Lee B., Theologidis I., Luna B. (2021). Environmental Spread of Antibiotic Resistance. Antibiotics.

[4] Schrader S.M., Vaubourgeix J., Nathan C. (2020). Biology of antimicrobial resistance and approaches to combat it. Sci. Transl. Med..

[5] Vidovic N., Vidovic S. (2020). Antimicrobial Resistance and Food Animals: Influence of Livestock Environment on the Emergence and Dissemination of Antimicrobial Resistance. Antibiotics.

[6] Ghosh C., Sarkar P., Issa R., Haldar J. (2019). Alternatives to Conventional Antibiotics in the Era of Antimicrobial Resistance. Trends Microbiol..

[7] Marquardt R.R., Li S. (2018). Antimicrobial resistance in livestock: Advances and alternatives to antibiotics. Anim. Front..

[8] Mookherjee N., Anderson M.A., Haagsman H.P., Davidson D.J. (2020). Antimicrobial host defence peptides: Functions and clinical potential. Nat. Rev. Drug Discov..

[9] Magana M., Pushpanathan M., Santos A.L., Leanse L., Fernandez M., Ioannidis A., Giulianotti M.A., Apidianakis Y., Bradfute S., Ferguson A.L. (2020). The value of antimicrobial peptides in the age of resistance. Lancet Infect. Dis..

[10] Hancock R.E., Haney E.F., Gill E.E. (2016). The immunology of host defence peptides: Beyond antimicrobial activity. Nat. Rev. Immunol..

[11] Wang G. (2014). Human antimicrobial peptides and proteins. Pharmaceuticals.

[12] Zhang G., Sunkara L.T. (2014). Avian antimicrobial host defense peptides: From biology to therapeutic applications. Pharmaceuticals.

[13] Cuperus T., Coorens M., van Dijk A., Haagsman H.P. (2013). Avian host defense peptides. Dev. Comp. Immunol..

[14] Wu J., Ma N., Johnston L.J., Ma X. (2020). Dietary Nutrients Mediate Intestinal Host Defense Peptide Expression. Adv. Nutr..

[15] Robinson K., Ma X., Liu Y., Qiao S., Hou Y., Zhang G. (2018). Dietary modulation of endogenous host defense peptide synthesis as an alternative approach to in-feed antibiotics. Anim. Nutr..

[16] Lyu W., Curtis A.R., Sunkara L.T., Zhang G. (2015). Transcriptional Regulation of Antimicrobial Host Defense Peptides. Curr. Protein Pept. Sci..

[17] Rodriguez-Carlos A., Jacobo-Delgado Y.M., Santos-Mena A.O., Rivas-Santiago B. (2021). Modulation of cathelicidin and defensins by histone deacetylase inhibitors: A potential treatment for multi-drug resistant infectious diseases. Peptides.

[18] Chen J., Zhai Z., Long H., Yang G., Deng B., Deng J. (2020). Inducible expression of defensins and cathelicidins by nutrients and associated regulatory mechanisms. Peptides.

[19] Gombart A.F., Borregaard N., Koeffler H.P. (2005). Human cathelicidin antimicrobial peptide (CAMP) gene is a direct target of the vitamin D receptor and is strongly up-regulated in myeloid cells by 1,25-dihydroxyvitamin D3. FASEB J..

[20] Zhang L., Lu L., Li S., Zhang G., Ouyang L., Robinson K., Tang Y., Zhu Q., Li D., Hu Y. (2016). 1,25-Dihydroxyvitamin-D3 Induces Avian beta-Defensin Gene Expression in Chickens. PLoS ONE.

[21] Sunkara L.T., Achanta M., Schreiber N.B., Bommineni Y.R., Dai G., Jiang W., Lamont S., Lillehoj H.S., Beker A., Teeter R.G. (2011). Butyrate enhances disease resistance of chickens by inducing antimicrobial host defense peptide gene expression. PLoS ONE.

[22] Zeng X., Sunkara L.T., Jiang W., Bible M., Carter S., Ma X., Qiao S., Zhang G. (2013). Induction of porcine host defense Peptide gene expression by short-chain Fatty acids and their analogs. PLoS ONE.

[23] Lyu W., Deng Z., Sunkara L.T., Becker S., Robinson K., Matts R., Zhang G. (2018). High Throughput Screening for Natural Host Defense Peptide-Inducing Compounds as Novel Alternatives to Antibiotics. Front. Cell. Infect. Microbiol..

[24] Hong Y.H., Song W., Lee S.H., Lillehoj H.S. (2012). Differential gene expression profiles of beta-defensins in the crop, intestine, and spleen using a necrotic enteritis model in 2 commercial broiler chicken lines. Poult. Sci..

[25] Mowbray C.A., Niranji S.S., Cadwell K., Bailey R., Watson K.A., Hall J. (2018). Gene expression of AvBD6-10 in broiler chickens is independent of AvBD6, 9, and 10 peptide potency. Vet. Immunol. Immunopathol..

[26] Zhang J.H., Chung T.D., Oldenburg K.R. (1999). A Simple Statistical Parameter for Use in Evaluation and Validation of High Throughput Screening Assays. J. Biomol. Screen..

[27] Zhang X.D. (2007). A new method with flexible and balanced control of false negatives and false positives for hit selection in RNA interference high-throughput screening assays. J. Biomol. Screen..

[28] Carlos-Reyes A., Lopez-Gonzalez J.S., Meneses-Flores M., Gallardo-Rincon D., Ruiz-Garcia E., Marchat L.A., Astudillo-de la Vega H., Hernandez de la Cruz O.N., Lopez-Camarillo C. (2019). Dietary Compounds as Epigenetic Modulating Agents in Cancer. Front. Genet..

[29] Beug H., von Kirchbach A., Doderlein G., Conscience J.F., Graf T. (1979). Chicken hematopoietic cells transformed by seven strains of defective avian leukemia viruses display three distinct phenotypes of differentiation. Cell.

[30] Liu L., Chen B., Qin S., Li S., He X., Qiu S., Zhao W., Zhao H. (2010). A novel histone deacetylase inhibitor Chidamide induces apoptosis of human colon cancer cells. Biochem. Biophys. Res. Commun..

[31] Perri F., Longo F., Giuliano M., Sabbatino F., Favia G., Ionna F., Addeo R., Della Vittoria Scarpati G., Di Lorenzo G., Pisconti S. (2017). Epigenetic control of gene expression: Potential implications for cancer treatment. Crit. Rev. Oncol. Hematol..

[32] Shvedunova M., Akhtar A. (2022). Modulation of cellular processes by histone and non-histone protein acetylation. Nat. Rev. Mol. Cell Biol..

[33] Ho T.C.S., Chan A.H.Y., Ganesan A. (2020). Thirty Years of HDAC Inhibitors: 2020 Insight and Hindsight. J. Med. Chem..

[34] Cochran A.G., Conery A.R., Sims R.J. (2019). Bromodomains: A new target class for drug development. Nat. Rev. Drug Discov..

[35] Schwalm M.P., Knapp S. (2022). BET bromodomain inhibitors. Curr. Opin. Chem. Biol..

[36] Ottosson H., Nylén F., Sarker P., Miraglia E., Bergman P., Gudmundsson G.H., Raqib R., Agerberth B., Strömberg R. (2016). Potent Inducers of Endogenous Antimicrobial Peptides for Host Directed Therapy of Infections. Sci. Rep..

[37] Deng Z., Wang J., Lyu W., Wieneke X., Matts R., Ma X., Zhang G. (2018). Development of a Cell-Based High-Throughput Screening Assay to Identify Porcine Host Defense Peptide-Inducing Compounds. J. Immunol. Res..

[38] Schauber J., Iffland K., Frisch S., Kudlich T., Schmausser B., Eck M., Menzel T., Gostner A., Luhrs H., Scheppach W. (2004). Histone-deacetylase inhibitors induce the cathelicidin LL-37 in gastrointestinal cells. Mol. Immunol..

[39] Sunkara L.T., Jiang W., Zhang G. (2012). Modulation of antimicrobial host defense peptide gene expression by free fatty acids. PLoS ONE.

[40] Myszor I.T., Parveen Z., Ottosson H., Bergman P., Agerberth B., Stromberg R., Gudmundsson G.H. (2019). Novel aroylated phenylenediamine compounds enhance antimicrobial defense and maintain airway epithelial barrier integrity. Sci. Rep..

[41] Sarker P., Banik A., Stromberg R., Gudmundsson G.H., Raqib R., Agerberth B. (2017). Treatment with Entinostat Heals Experimental Cholera by Affecting Physical and Chemical Barrier Functions of Intestinal Epithelia. Antimicrob. Agents Chemother..

[42] Adcock I.M. (2007). HDAC inhibitors as anti-inflammatory agents. Br. J. Pharmacol..

[43] Zucconi B.E., Luef B., Xu W., Henry R.A., Nodelman I.M., Bowman G.D., Andrews A.J., Cole P.A. (2016). Modulation of p300/CBP Acetylation of Nucleosomes by Bromodomain Ligand I-CBP112. Biochemistry.

[44] Pandit B., Royzen M. (2022). Recent Development of Prodrugs of Gemcitabine. Genes.

[45] Crouwel F., Simsek M., Mulder C.J., Buiter H.J., De Boer N.K. (2020). Thioguanine Therapy in Inflammatory Bowel Diseases. A Practical Guide. J. Gastrointest. Liver Dis..

[46] Rugo H.S., Jacobs I., Sharma S., Scappaticci F., Paul T.A., Jensen-Pergakes K., Malouf G.G. (2020). The Promise for Histone Methyltransferase Inhibitors for Epigenetic Therapy in Clinical Oncology: A Narrative Review. Adv. Ther..

[47] Whitmore M.A., Li H., Lyu W., Khanam S., Zhang G. (2022). Epigenetic Regulation of Host Defense Peptide Synthesis: Synergy between Histone Deacetylase Inhibitors and DNA/Histone Methyltransferase Inhibitors. Front. Immunol..

[48] Ghate N.B., Kim S., Spiller E., Kim S., Shin Y., Rhie S.K., Smbatyan G., Lenz H.J., Mumenthaler S.M., An W. (2021). VprBP directs epigenetic gene silencing through histone H2A phosphorylation in colon cancer. Mol. Oncol..

[49] Rath N.C., Parcells M.S., Xie H., Santin E. (2003). Characterization of a spontaneously transformed chicken mononuclear cell line. Vet. Immunol. Immunopathol..

[50] Wang J., Lyu W., Zhang W., Chen Y., Luo F., Wang Y., Ji H., Zhang G. (2021). Discovery of natural products capable of inducing porcine host defense peptide gene expression using cell-based high throughput screening. J. Anim. Sci. Biotechnol..

[51] Yang Q., Whitmore M.A., Robinson K., Lyu W., Zhang G. (2021). Butyrate, Forskolin, and Lactose Synergistically Enhance Disease Resistance by Inducing the Expression of the Genes Involved in Innate Host Defense and Barrier Function. Antibiotics.

[52] Robinson K., Yang Q., Li H., Zhang L., Aylward B., Arsenault R.J., Zhang G. (2021). Butyrate and Forskolin Augment Host Defense, Barrier Function, and Disease Resistance without Eliciting Inflammation. Front. Nutr..

[53] Yang Q., Fong L.A., Lyu W., Sunkara L.T., Xiao K., Zhang G. (2021). Synergistic Induction of Chicken Antimicrobial Host Defense Peptide Gene Expression by Butyrate and Sugars. Front. Microbiol..

[54] Yang Q., Chen B., Robinson K., Belem T., Lyu W., Deng Z., Ramanathan R., Zhang G. (2022). Butyrate in combination with forskolin alleviates necrotic enteritis, increases feed efficiency, and improves carcass composition of broilers. J. Anim. Sci. Biotechnol..

[55] Schmittgen T.D., Livak K.J. (2008). Analyzing real-time PCR data by the comparative C(T) method. Nat. Protoc..

